# Diagnostic Evaluation of Small Intestinal Microbial Overgrowth: A Cross-Sectional Comparison of Glucose and Lactulose Breath Tests

**DOI:** 10.3390/jcm14248920

**Published:** 2025-12-17

**Authors:** Giulia Scalese, Luca Spina, Lucia Gallucci, Alessandra Cesarini, Emanuela Ribichini, Maddalena Diofebi, Ivan Tattoli, Lucia Pallotta, Anna Citarella, Carola Severi

**Affiliations:** 1 Department of Translational and Precision Medicine, Sapienza University of Rome, Piazzale Aldo Moro 5, 00185 Rome, Italy; giulia.scalese@uniroma1.it (G.S.); alessandra.cesarini@uniroma1.it (A.C.); emanuela.ribichini@uniroma1.it (E.R.); ivan.tattoli@uniroma1.it (I.T.); lucia.pallotta@uniroma1.it (L.P.); 2 Department of Statistical Sciences, Sapienza University of Rome, Piazzale Aldo Moro 5, 00185 Rome, Italy; lucia.gallucci@uniroma1.it; 3 LinkHealth Health Economics, Outcomes & Epidemiology S.R.L., 80143 Naples, Italy

**Keywords:** Small Intestinal Bacterial Overgrowth (SIBO), Intestinal Methanogen Overgrowth (IMO), breath testing, hydrogen, methane, Archaea, gut microbiota

## Abstract

**Background/Objectives**: Small intestinal microbial overgrowth (SIMO), including both small intestinal bacterial overgrowth (SIBO) and intestinal methanogen overgrowth (IMO), is commonly diagnosed using non-invasive breath tests, whose diagnostic performance and criteria remain inconsistent. This study aimed to assess SIMO prevalence using lactulose (LBT) and glucose breath tests (GBT), compare their diagnostic yields for SIBO and IMO, analyze associated gas profiles, clinical features, risk factors, and evaluate the diagnostic accuracy of a simplified fasting methane criterion for IMO. **Methods**: Cross-sectional study conducted on 564 outpatients (75.7% female) with suspected SIMO. Patients underwent LBT (n = 275), GBT (n = 289), or both (n = 47). **Results**: SIMO was diagnosed in 26.8% of patients. LBT identified significantly more SIMO than GBT (37.5% vs. 16.6%, *p* < 0.01), particularly for SIBO (24.4% vs. 4.8%, *p* < 0.01), while IMO detection was comparable (9.8% vs. 10.7%). Mixed overgrowth (dual SIBO/IMO positivity) showed a borderline trend favoring LBT. Methane peaks occurred significantly earlier than hydrogen in both BTs. Clinical symptoms did not significantly differ between SIMO subtypes or between test-positive and test-negative groups. The simplified fasting methane criterion showed limited diagnostic accuracy for IMO making it inadequate as a standalone diagnostic tool, requiring further validation before clinical implementation. **Conclusions**: GBT is the more reliable test for SIMO diagnosis due to LBT’s lower specificity. Clinical symptoms alone were not predictive of SIMO subtypes, while the different gas profile suggests a distinct spatial distribution of microbial populations with a higher proximal concentration of methanogenic Archaea.

## 1. Introduction

Breath tests (BTs) are widely used, non-invasive, and cost-effective alternatives to duodenal/jejunal aspirate cultures for diagnosing small intestinal bacterial overgrowth (SIBO) [1,2,3]. Because human cells do not produce hydrogen (H_2_) or methane (CH_4_), their presence in exhaled breath reflects microbial carbohydrate fermentation in the gut [4]. Currently, glucose (GBT) and lactulose breath tests (LBT) are commonly used modalities for detecting SIBO.

Recent advances have identified intestinal methanogen overgrowth (IMO) as a distinct clinical entity, characterized by the excessive proliferation in the gut of methanogenic Archaea, especially Methanobrevibacter smithii [5]. Archaea are unicellular microorganisms that phenotypically resemble bacteria but belong to a separate domain with a distinct metabolism, producing CH_4_ through the conversion of H_2_ [6]. Since culturing Archaea is technically challenging in most clinical microbiology laboratories, aspirate testing is not viable for diagnosing IMO. As a result, mandatory CH_4_ measurement in BTs has become essential to distinguish between SIBO and IMO.

Both conditions share common risk factors, including anatomical abnormalities, impaired motility, and reduced bacteriostatic activity of the small intestine [7,8]. They clinically present with overlapping non-specific gastrointestinal symptoms, such as bloating, flatulence, abdominal discomfort, and altered bowel habits [9]. However, diarrhea is more strongly associated with SIBO [10], whereas constipation is the hallmark symptom of IMO [5,11].

Differentiating between SIBO and IMO is critical for selecting appropriate treatment. IMO often requires combination antibiotic therapy, most notably neomycin and rifaximin [10], while SIBO can typically be managed with monotherapy, usually rifaximin [12]. These differences support the view that SIBO and IMO should be considered two distinct forms of small intestinal microbial overgrowth (SIMO), which can only be reliably distinguished via BTs. Despite the clinical importance, universal diagnostic standards remain lacking, with various international guidelines proposing different cutoff values [1,13].

This study aimed to assess the prevalence of SIMO using LBT and GBT, compare their diagnostic yields for SIBO and IMO, and explore the associations between SIMO subtypes with specific gas profiles, clinical symptoms, and risk factors. A secondary aim was to evaluate the diagnostic performance of a simplified criterion for IMO diagnosis based solely on basal fasting CH_4_ levels [14].

## 2. Materials and Methods

This retrospective cross-sectional study included all 585 consecutive adult outpatients who were referred to the Gastroenterology Unit’s breath test service at Policlinico Umberto I, Sapienza University of Rome, between January 2022 and December 2024. The study followed the principles of the Declaration of Helsinki (6th revision, 2008) and received ethical approval (no. 7643) from the CET–Comitato Etico Territoriale Lazio Area 1 initially on 28 June 2024, which authorized the retrospective enrollment of all patients accessing the breath test service from January 2022 up to the date of authorization. Afterwards, the study period was extended until June 2025 through an amendment to the approval on 5 February 2025. Written informed consent was obtained from all participants.

Inclusion criteria included adult outpatients aged 16 to 80 years referred to the breath test service for GBT and/or LBT. Exclusion criteria included patients younger than 16 or older than 80 years, refusal to participate, inability to complete the test, hospitalization, or the presence of any clinical contraindication to participating in the protocol (e.g., active or non-remitted neoplasms, chronic infections, primary or acquired immunodeficiency, alcoholism, NYHA class IV heart failure, and psychiatric diseases). Additional exclusion criteria were pregnancy or breastfeeding. Before testing, all patients were asked to abstain from antibiotics for the four preceding months. Additionally, laxatives, antidiarrheals, prokinetics, and spasmolytics were discontinued at least 48 h before the tests [1]. Probiotic intake was discontinued two weeks before the BT. Patients received detailed dietary instructions to limit their fiber intake on the day before the procedure, avoiding foods rich in fermentable oligosaccharides, disaccharides, monosaccharides, and polyols (FODMAPs), such as fruits, vegetables, grains, and bran cereals. All patients underwent BTs in the morning after overnight fasting (≥8 h) and mouth cleaning. Smoking and exercise at least 2 h before and during the test were not allowed, as hyperventilation can alter BT results. Those who did not comply were excluded.

Demographic data (including age, weight, and height) were collected on the day of testing. After the study had commenced, it became necessary to additionally collect anamnestic information on risk factors and clinical data regarding symptomatology to allow for a more accurate characterization of the diagnoses, as the participants were outpatients for whom the BTs had been requested by other physicians. Consequently, symptom data were available for 287 patients (142 from LBT; 145 from GBT), while medical histories were obtained from 275 patients (138 from LBT; 137 from GBT). Known SIMO risk factors included the following: (I) surgical history (intestinal, ascending colon, or gastric resections); (II) prior abdominal radiotherapy; and (III) predisposing clinical conditions (diabetes mellitus, systemic sclerosis, amyloidosis, chronic atrophic gastritis, celiac disease, Crohn’s disease, chronic pancreatitis, hepatic cirrhosis, stage III–IV chronic kidney disease, fibromyalgia) [1]. Clinical symptoms were assessed using a Rome IV-based questionnaire [15] and categorized as dyspepsia, abdominal pain, alternating bowel habits, constipation, diarrhea, and flatulence.

LBT was performed by administering 10 g of lactulose, with breath samples collected every 15 min for up to 240 min. GBT used 50 g of glucose diluted in 250 mL of water, with samples collected for up to 120 min [1,16]. In patients undergoing both tests, LBT and GBT were conducted on separate days. H_2_ and CH_4_ levels were measured using the Lactotest 202–Xtend (Medical Electronic Construction R&D, Brussels, Belgium). The two BTs were interpreted according to the diagnostic cut-off values reported in the current European Guidelines [1]. For the diagnosis of SIBO, an increase in hydrogen (H_2_) concentration of at least (≥) 20 parts per million (ppm) above baseline within 90 min from the beginning of the test was considered diagnostic for LBT, whereas a rise in H_2_ concentration ≥ 10 ppm any time during the test was used for GBT. For IMO, an increase in methane (CH_4_) levels ≥ 10 ppm above baseline within 90 min from the beginning of the test was considered diagnostic for LBT, while a rise in CH_4_ concentration ≥ 10 ppm any time during the test was used for GBT. Positivity to both H_2_ and CH_4_ cut-off values was defined as mixedovergrowth. A schematic representation of the diagnostic criteria used in the study is provided in Figure S1.

To evaluate the possible application of the simplified breath-testing protocol for diagnosing IMO, the standard diagnostic criteria reported in the European Guidelines were compared with the recent ones proposed by an American multicenter group, which considers the only basal fasting CH_4_ concentration ≥ 10 ppm [14].

For statistical analysis, patients were grouped by the type of BT. All analyses were conducted using R version 4.4.3. Quantitative variables were summarized as medians with interquartile ranges (IQRs). Group comparisons for quantitative variables were performed using the Mann–Whitney U test for two groups and the Kruskal–Wallis test for comparisons involving more than two groups, as these non-parametric tests are appropriate for data that do not follow a normal distribution. The Chi-squared test was used to compare proportions between independent groups; when expected cell counts were low, Fisher’s exact test was applied instead. The McNemar test was used for paired categorical data. Multiple comparisons were performed using Bonferroni correction. A *p*-value of <0.05 was considered statistically significant.

## 3. Results

### 3.1. Population Characteristics and Test Distribution

**Figure 1:**
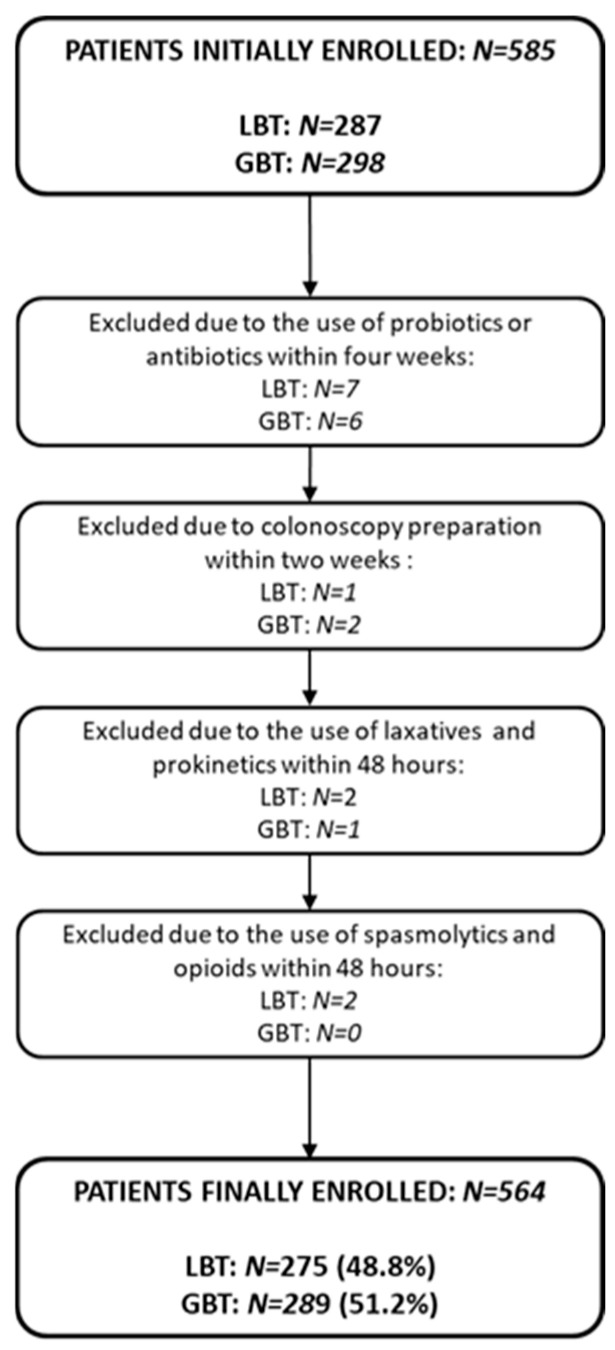
Study flowchart illustrating patient selection. Abbreviations: LBT: lactulose breath test; GBT: glucose breath test.

Out of 585 patients initially enrolled, 564 were included after excluding 21 for non-compliance with preparation protocols (Figure 1).

**Figure 2:**
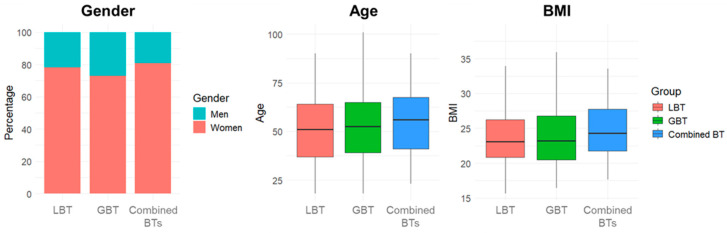
Demographic and anthropometric characteristics distribution. Abbreviations: BTs: breath tests; LBT: lactulose breath test; GBT: glucose breath test; BMI: body mass index.

Among these, 275 (48.8%) underwent LBT, 289 (51.2%) GBT, and 47 (8.3%) completed both. The majority were female (75.7%), with a median age of 52 years (IQR: 38–65 years) and a median BMI of 23.15 kg/m^2^ (IQR: 20.70–26.74 kg/m^2^). Demographic and anthropometric characteristics for each group are reported in Table S1. A chi-square test performed on the gender proportions (*p* = 0.14), and a Kruskal–Wallis test conducted on BMI values (*p* = 0.50) and age distribution (*p* = 0.21), revealed no significant differences between groups, concluding that the samples were comparable and no confounding factors could affect the comparisons between the groups (Figure 2).

The principal BTs prescribers were gastroenterologists (59.1%), both for LBT and GBT (60.8% and 57.5%, respectively), with no preference for either (*p* = 0.44). General practitioners represented the second most frequent prescribers (25.4%), with a significant preference for GBT (*p* < 0.01), while allergists/immunologists (13.0%) and nutritionists (0.8%) showed a significant preference for LBT (*p* = 0.01 and *p* = 0.04, respectively).

### 3.2. Prevalence and Detection of Small Intestinal Microbial Overgrowth (SIMO)

SIMO, defined as a positive breath test for hydrogen (H_2_), methane (CH_4_), or both, was detected in 26.8% (151/564) of patients. SIBO (H_2_-positive) was statistically more prevalent than IMO (CH_4_-positive), at 14.4% vs. 10.3% (*p* = 0.04), while mixed overgrowth (positive both H_2_ and CH_4_) occurred in 2.1% (Table 1). The positivity rate for SIBO was significantly higher in the LBT group than in the GBT group (24.4% vs. 4.8%, respectively; *p* < 0.01), though both tests detected IMO at similar rates (9.8% vs. 10.7%; *p* = 0.93). The corrected significance level applying Bonferroni correction was 0.01 (0.05/5).

**Figure 3:**
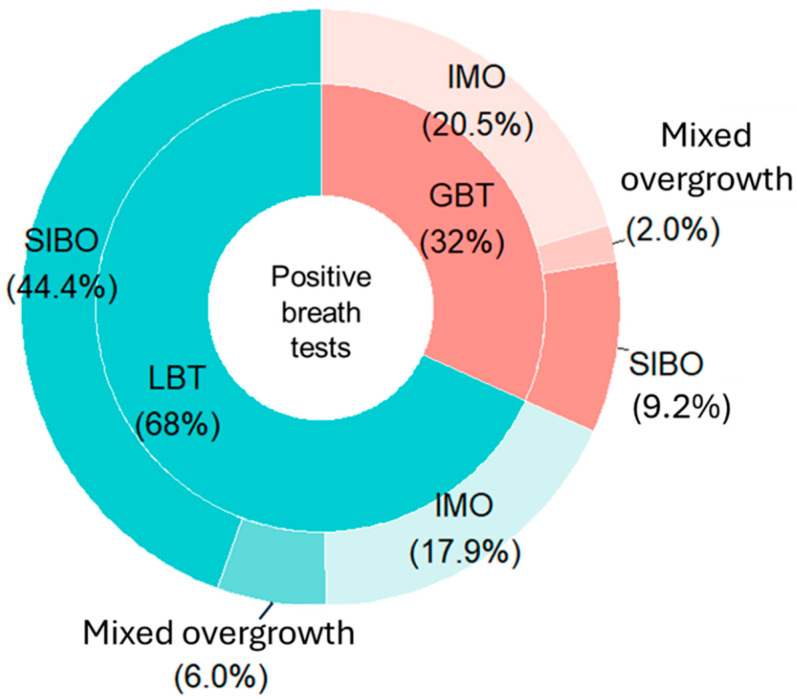
Distribution of small intestinal bacterial overgrowth, intestinal methanogen overgrowth, and mixed overgrowth diagnoses for each breath test among positive patients. Abbreviations: LBT: lactulose breath test; GBT: glucose breath test; SIBO: Small Intestinal Bacterial Overgrowth; IMO: Intestinal Methanogen Overgrowth.

When differentiating between the two overgrowth subtypes in SIMO-positive patients, SIBO was still identified by LBT in more than twice as many cases as GBT, being the most frequently diagnosed condition in patients undergoing LBT. By contrast, IMO was the predominant diagnosis in the GBT group. Notably, despite these differences in distribution compared to the overall sample, the proportion of IMO diagnoses was relatively similar between the two tests (17.9% in LBT and 20.5% in GBT) (Figure 3).

In the subgroup of 47 patients who underwent both tests, SIMO prevalence was significantly higher (48.9%) compared to the overall cohort (26.8%) (*p* < 0.01). Among the diagnoses of SIMO, 74.0% were identified exclusively by LBT, and 13.0% exclusively by GBT. Only 8.7% of cases showed concordant results between the two breath tests (both LBT and GBT tested positive for IMO), whereas in 4.3% of cases the diagnosis was discordant (SIBO with LBT, and IMO with GBT) (Table S2). However, due to the small sample size of this subgroup, the κ-concordance index could not be calculated.

### 3.3. Breath Test Gas Profiles

**Figure 4:**
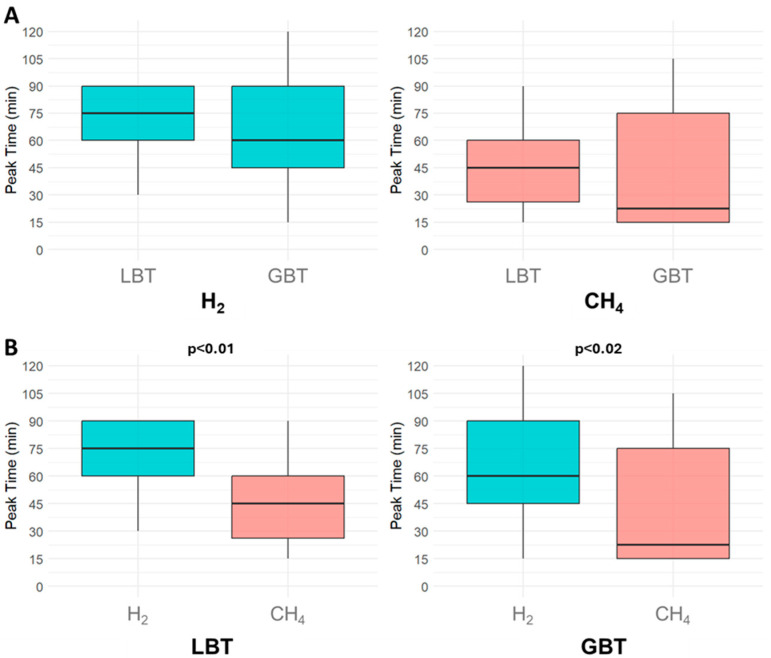
Comparison of gas patterns in small intestinal bacterial overgrowth and intestinal methanogen overgrowth. (**A**) shows the differences in hydrogen and methane median diagnostic peak time between the lactulose breath test and the glucose breath test. (**B**) shows the comparison of the median diagnostic peak times between the two gases within each breath test individually. Abbreviations: H_2_: hydrogen; CH_4_: methane; LBT: lactulose breath test; GBT: glucose breath test.

Although not statistically significant, H_2_ and CH_4_ diagnostic peaks tended to appear later in LBT than GBT. Specifically for SIBO, the median H_2_ diagnostic peak occurred at 75 min (IQR: 60–90 min) in positive LBT, compared to 60 min (IQR: 45–90 min) in positive GBT (*p* = 0.20). For IMO, the median CH_4_ peak occurred at 45 min (IQR: 26.25–60 min) in positive LBT and at 22.50 min (IQR: 15–75 min) in positive GBT (*p* = 0.40) (Figure 4A). Within each test, CH_4_ peaks occurred significantly earlier than H_2_ peaks (*p* < 0.01 for LBT, *p* = 0.02 for GBT), suggesting possible spatial differences in microbial colonization (Figure 4B). After applying the Bonferroni correction (α = 0.0125), the CH_4_–H_2_ peak difference remained significant in the LBT (*p* < 0.01), whereas the effect in the GBT (*p* = 0.02) fell just short of the corrected significance level and approached significance.

### 3.4. Clinical Presentation and Risk Factors

**Figure 5:**
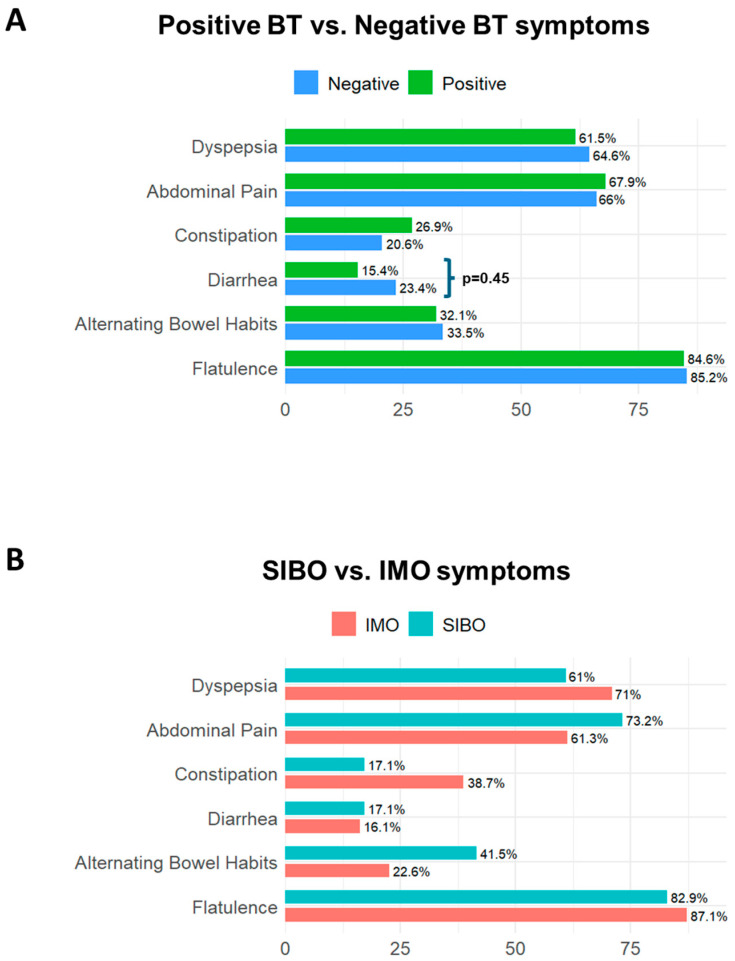
Prevalence of symptoms in small intestinal microbial overgrowth. (**A**) illustrates the differences in symptoms between patients with negative and positive breath tests; diarrhea was statistically higher in patients with negative breath tests than in those with positive ones (*p* = 0.045). (**B**) illustrates the differences in symptoms between patients with Small Intestinal Bacterial Overgrowth (SIBO) and those with Intestinal Methanogen Overgrowth (IMO); no single symptom reliably distinguished between SIBO and IMO. Abbreviations: BT: breath test; SIBO: Small intestinal Bacterial Overgrowth; IMO: Intestinal Methanogen Overgrowth.

The prevalence of symptoms did not differ between patients with positive versus negative BTs. Flatulence, dyspepsia and abdominal pain were the most frequently reported symptoms, but none clearly distinguished the positive from negative results. Diarrhea was interestingly more prevalent among SIMO-negative patients (*p* = 0.045), whereas constipation was more common in IMO patients (*p* = 0.06), aligning with the literature [11,17,18,19] (Figure 5A,B, Tables S3 and S4). The corrected significance level applying Bonferroni correction for the symptom analysis was 0.008. Given this adjusted threshold, these findings should be interpreted with caution.

Within the group of patients with available clinical history, 22.5% (62/275) had medical conditions predisposing to SIMO, mainly chronic atrophic gastritis (n = 36) and fibromyalgia (n = 16), 2.2% (6/275) had surgical risk factors, and 0.3% (1/275) underwent abdominal radiotherapy. However, none of these conditions were significantly associated with SIMO.

### 3.5. Simplified Diagnostic Criteria for IMO

Applying the simplified criterion for IMO, namely the fasting CH_4_ concentration ≥ 10 ppm [14], the overall IMO positivity rate increased to 18.4% (104/564), compared to 12.4% (70/564) identified by the standard diagnostic approach. Detection rate rose by 41.7% in the LBT group (13.1% versus 18.5%, respectively) and by 55.9% in the GBT group (11.8% versus 18.3%, respectively). However, this increase came at the expense of diagnostic accuracy. Compared to European standard guidelines [1], the simplified criterion reduced specificity and PPV in both test groups. In LBT, sensitivity (83.3%) and NPV (97.3%) remained high, but specificity dropped to 91.2% and PPV to 57.4%. In GBT, specificity fell to 85.5%, sensitivity to 47.1%, and PPV to 30.2%, suggesting a high rate of both false positives and negatives (Table 2).

## 4. Discussion

This study confirms a SIMO prevalence of approximately 30%, with SIBO being more common than IMO—consistent with previous reports [20,21]. In current clinical practice, LBT and GBT are extensively used by various healthcare professionals for diagnosing SIMO, primarily due to their availability, safety, and cost-effectiveness [3]. Nonetheless, the choice of test often remains at the discretion of the prescriber. However, in line with previous studies [22,23], our findings reveal significant differences in the detection rates of the two SIMO subtypes depending on the test used. Despite the widespread use of BTs by both gastroenterologists and non-specialists, this study emphasizes the importance of selecting diagnostic tools based on their performance and accuracy, rather than convenience or clinical habit. This is particularly crucial to ensure accurate differentiation between SIBO and IMO and to prevent the inappropriate use of antibiotics [24].

In particular, LBT yielded a higher positivity rate for SIBO compared to GBT. This likely reflects LBT’s lower specificity and higher false positive rate, possibly due to interindividual variability in intestinal transit time and the properties of lactulose itself. Lactulose, in fact, is a disaccharide that is not absorbed in the human small intestine and is fermented only in the colon. Therefore, its fermentation time is influenced by the orocecal transit time (OCTT), which refers to the time required for ingested material to travel from the mouth to the cecum. Consequently, conditions that accelerate OCTT—such as diarrhea-predominant IBS or the use of lactulose itself—may lead to earlier fermentation, producing an early H_2_ peak and thus resulting in a false positive SIBO diagnosis [1,25]. Previous meta-analyses [2] reported higher sensitivity and specificity for GBT, and current guidelines recommend it as the preferred diagnostic tool for SIBO [26,27,28]. However, it is important to note that the lower specificity of LBT reported in our study is inferred rather than directly measured, as duodenal/jejunal aspirate cultures—the gold standard for diagnosing SIBO—were not available. Therefore, the apparent excess of LBT-positive results cannot be quantified in terms of actual false positives. Moreover, since the two BTs were largely performed in different patient populations, cross-test comparisons in this study are inherently indirect and may reflect differences in referral patterns.

Despite this aspect, the higher sensitivity and specificity of GBT seem to be further supported by data from the subgroup of patients who underwent both tests. Although the sample size was insufficient for statistical analysis, the prevalence of SIMO in this subgroup was notably higher (48.9%) compared to the overall study population (26.8%), and the majority of these cases were identified primarily by LBT, reinforcing the observation of its lower specificity and higher false positive rate. Considering IMO, both tests performed similarly, suggesting interchangeability for CH_4_ detection. However, the poor concordance between LBT and GBT results in patients undergoing both tests (only two had concordant IMO diagnoses) suggests limited interchangeability between them [29]. However, this subgroup of patients was small and underpowered, because comparing the two tests in the same patient is very difficult in real clinical practice. Indeed, clinicians usually request only one of the two BTs, and patients often refuse to undergo both because the tests are lengthy and require patient preparation. Therefore, the observed poor concordance between the two methods should be interpreted with caution.

Despite GBT’s relative advantages, both BTs remain suboptimal diagnostic tools [30,31], likely due to their widespread and often indiscriminate use in clinical practice—even in the absence of a clear clinical indication. According to the European Guidelines, LBT and GBT should be prescribed only to those patients who present specific risk factors–such as diabetes mellitus or systemic sclerosis–and after a careful differential evaluation of related symptoms. By contrast, a growing trend involves attributing virtually any nonspecific gastrointestinal symptom to SIBO, contributing to diagnostic ambiguity. In recent years, even extraintestinal manifestations–such as headaches, mood disturbances, general malaise, skin disorders, hepatic changes, and arthralgia–have been increasingly associated with SIBO [32,33]. This broad and often uncritical clinical interpretation risks diluting the original definition of SIBO, which historically referred to microbial overgrowth in the small intestine, typically presenting with clear clinical signs, particularly those related to malabsorption [24]. In our study, the presence of predisposing conditions did not significantly increase the likelihood of SIMO diagnosis.

Similarly, no gastrointestinal symptom—except for a slightly higher prevalence of diarrhea among SIMO-negative patients—reliably distinguished SIMO-positive from SIMO-negative patients. Interestingly, diarrhea was slightly less prevalent among positive patients, but this data was not confirmed by the multiple comparisons, suggesting that this finding is likely a chance observation rather than a biologically meaningful difference. Moreover, the overall high prevalence of diarrhea in our cohort likely reflects the functional nature of gastrointestinal symptoms in the population for whom BTs are typically ordered, as diarrhea is often one of the primary reasons for requesting this type of test. Moreover, no gastrointestinal symptom can differentiate between SIBO and IMO, except for a borderline association between constipation and IMO. The lack of statistical significance may be explained by the small sample size and the exploratory nature of the symptom data analysis. Since the literature documented a significant association between these two conditions [11,17,18,19], we assume that a much larger sample could have aligned with these results.

Although these findings are limited by the low prevalence of comorbidities and the specific clinical setting, they nonetheless support a more judicious use of BTs: breath testing should be reserved for patients with strong clinical indications, following comprehensive clinical assessment and appropriate exclusion of alternative diagnoses.

In addition to the uncertainty surrounding clear indications for their prescription, equally significant problems exist regarding the interpretation of results. In practice, despite the frequent use of these tests in both general and specialized medical settings, many healthcare professionals are not well-acquainted with BTs interpretation. Additionally, diagnostic criteria remain a challenge, varying significantly across different guidelines and expert consensus statements [1,34]. A recent proposal from a U.S.-based research group introduced a novel diagnostic criterion for IMO, emphasizing the role of fasting methane levels, with a fasting CH_4_ level of ≥10 ppm being indicative of IMO [14]. This approach reflects the growing recognition of baseline CH_4_ production as a reliable marker of methanogen overgrowth, offering both a potentially more straightforward and standardized diagnostic threshold for clinical use and a significant shortening of BTs, reducing laboratory workload and costs. The simplified fasting CH_4_ criterion proposed by a U.S. study was promising in terms of ease and efficiency [14], but its diagnostic accuracy was not replicated here. Although sensitivity and NPV remained high in the LBT group, specificity and PPV decreased significantly. In GBT, performance dropped across all parameters, including sensitivity, specificity, and PPV. This discrepancy may be due to differences in sample size, patient population, or laboratory methodology. Nevertheless, our findings caution against replacing standard diagnostic criteria with simplified ones without further validation.

Finally, turning to the intrinsic characteristics of SIBO and IMO using both BTs, our study also sought to analyze the profiles in diagnostic peak times of H_2_ and CH_4_ concentrations, both separately and in combination. Our analysis of gas peak times did not support prior hypotheses that GBT is more sensitive to proximal SIBO (due to the proximal absorption of the glucose), and LBT better detects distal overgrowth [35], because the timing of H_2_ and CH_4_ increase did not significantly differ between the two breath tests. Instead, the statistically earlier occurrence of CH_4_ peaks compared to H_2_ peaks, both in LBT and GBT, may indicate a distinct spatial distribution of microbial populations, with methanogenic Archaea being more concentrated proximally and hydrogen-producing bacteria colonizing more distal segments of the small intestine, presumably in the ileum, where SIBO can, in the most severe cases, reduce vitamin B12 absorption [20]. Therefore, rather than reflecting differing diagnostic effectiveness between LBT and GBT based on overgrowth location, this pattern points to a potential variation in microbial colonization. However, the microbiological profiling of intestinal overgrowth is unfortunately still problematic, as the current diagnostic gold-standard for SIMO, the duodenal or jejunal aspirate (which should allow to obtain microbiological samples), raises many concerns, including the risk of sample contamination from oral or pharyngeal microbiota, and uncertainty regarding the diagnostic cut-off values (10^3^ vs. 10^5^ CFU/mL) [4,13], making the diagnosis quite ambiguous. In this context, the genomic and metabolomic profiling of the intestinal microbiota represents a promising frontier for the characterization of SIMO. However, this approach is also challenged by significant issues, including methodological heterogeneity, a lack of standardized protocols, and high costs [36]. Further research is essential to clarify the role of microbial community structure in SIMO and to understand its implications for the interpretation and optimization of BTs diagnostics [36]. In the meantime, a prudent approach would be to select the type of BT (glucose vs. lactulose) based on the suspected form of intestinal overgrowth. For example, in patients with constipation, or suspected involvement of the proximal small intestine, both GBT and LBT are equally indicated as they demonstrate comparable performance in detecting intestinal methanogen overgrowth (IMO). Conversely, in cases characterized by malabsorption or systemic conditions associated with accelerated intestinal transit or anatomical abnormalities, GBT may be more appropriate for SIMO, due to its higher specificity and lower rate of false positive results.

Key strengths of this study include a large sample size and comprehensive access to analytical records. However, the study’s retrospective and non-randomized design, along with variability in test indications and prescriber expertise, limits its generalizability. In terms of limitations, the sample is predominantly composed of middle-aged women, so the results may not be generalizable to other demographic groups. However, the study population is representative of a real clinical practice setting, where we observed that BTs are mainly requested to investigate nonspecific symptoms compatible with functional gastrointestinal disorders, mostly reported in the female population. Moreover, incomplete data on symptoms and risk factors for a subset of patients potentially led to an underestimation of associations, and the very small sample size of patients performing both BTs limited a paired comparison between LBT and GBT, thereby restricting the robustness of the observed poor concordance between the two methods.

We believe that forthcoming prospective or interventional studies should evaluate the role of glucose and lactulose BTs as practical, low-cost, and non-invasive screening tools to help identify patients with a higher probability of small intestinal dysbiosis or motility disorders, who could then be selectively referred for second-level diagnostic procedures. These include duodenal/jejunal aspirate culture—considered the reference method for the diagnosis of SIBO but invasive and resource-intensive—and scintigraphic assessment of OCTT, which although informative, involves radiation exposure and higher costs. Importantly, the successful implementation of such a stepwise diagnostic strategy will require further refinement and standardization of BT methodology. Enhancing reproducibility, harmonizing cut-off values, and improving measurement protocols would strengthen the interpretability of the test results and increase their clinical utility. Future studies should therefore assess whether a more standardized BT approach, used as an initial screening tool, can improve diagnostic accuracy, reduce unnecessary invasive procedures, and better stratify patients for targeted interventions. In this sense, concise prospective validation would help clarify the clinical utility and cost-effectiveness of integrating BTs into routine diagnostic pathways.

In conclusion, GBT is preferred over LBT due to its superior specificity and reliability. Methane measurement is essential for accurately identifying intestinal methanogen overgrowth (IMO), but although the simplified CH_4_ criterion (fasting CH_4_ ≥ 10 ppm) may streamline testing, it increases IMO detection at the expense of specificity and therefore should not replace validated diagnostic standards without further evidence. Although symptoms alone cannot distinguish SIBO from IMO, clinical presentation and specific diagnostic suspicion may guide the type of test to prescribe. In addition, the distinct timing of hydrogen (H_2_) and methane (CH_4_) peaks suggests different microbial localizations within the small intestine, offering insights into SIMO subtypes. These findings highlight the need for ongoing refinement in the clinical use of breath tests.

## Figures and Tables

**Table 1:** Prevalence of small intestinal microbial overgrowth in lactulose breath tests, glucose breath test and in the total sample classified in small intestinal bacterial overgrowth, intestinal methanogen overgrowth and mixed overgrowth, based on the gas exhaled.

	Lactulose Breath Test (n = 275)	Glucose Breath Test (n = 289)	*p*-Value	Total (n = 564)
Negatives	172 (62.5%)	241 (83.4%)	<0.01 †	413 (73.2%)
Positives	103 (37.5%)	48 (16.6%)	<0.01 †	151 (26.8%)
SIBO (peak in H_2_)	67 (24.4%)	14 (4.8%)	<0.01 †	81 (14.4%)
IMO (peak in CH_4_)	27 (9.8%)	31 (10.7%)	0.93 †	58 (10.3%)
Mixed overgrowth (dual peak in H_2_ and CH_4_)	9 (3.3%)	3 (1.1%)	0.09 ‡	12 (2.1%)

†: calculated using the chi-square test. ‡: calculated using Fisher’s exact test. Abbreviations: SIBO: Small Intestinal Bacterial Overgrowth; IMO: Intestinal Methanogen Overgrowth; H_2_: hydrogen; CH_4_: methane.

**Table 2:** Diagnostic performance of the simplified criterion for diagnosing Intestinal Methanogen Overgrowth compared to standard criteria.

	Positivity Rate	Specificity	Sensitivity	PPV	NPV
Lactulose breath test	18.5% (95%CI: 14.1–23.7%)	91.2% (95%CI: 86.9–94.5%)	83.3% (95%CI: 67.2–93.6%)	57.4% (95%CI: 43.2–70.8%)	97.3% (95%CI: 94.3–99%)
Glucose breath test	18.3% (95%CI: 14–23.3%)	85.5% (95%CI: 80.6–89.6%)	47.1% (95%CI: 29.8–64.9%)	30.2% (95%CI: 18–44.3%)	92.4% (95%CI: 88.2–95.4%)
Total	18.4% (95%CI: 15.3–21.9%)	88.6% (95%CI: 85.1–91%)	65.7% (95%CI: 53.4–76.6%)	44.2% (95%CI: 34.5–54.3%)	94.8% (95%CI: 92.3–96.6%)

Abbreviations: PPV: positive predictive value; NPV: negative predictive value; CI: confidence interval.

## Data Availability

The data that support the findings of this study are available from the corresponding author upon reasonable request.

## Supplementary Materials

The following supporting information can be downloaded at: https://www.mdpi.com/article/10.3390/jcm14248920/s1, Table S1: Demographic and anthropometric characteristics of the study population; Table S2: Prevalence of Small Intestinal Bacterial Overgrowth, Intestinal Methanogen Overgrowth and mixed-type Overgrowth in patients who underwent both lactulose and glucose breath test; Table S3: Differences in the prevalence of symptoms between negative and positive patients in the lactulose and glucose breath test subgroups; Table S4: Differences in the prevalence of symptoms between patients with SIBO and those with IMO in the lactulose and glucose breath test subgroups. Figure S1. Graphic representation of the interpretation of the gas curves in lactulose breath test (LBT) and in glucose breath test (GBT). In LBT, the cut-off was defined as an increase of at least (≥)20 parts per million (ppm) in H_2_ (SIBO) or ≥10 ppm in CH_4_ (IMO) above baseline by 90 min, while GBT was considered positive for SIMO with an increase of ≥10 ppm in H_2_ (SIBO) or CH_4_ (IMO) above baseline. Figure (A,B) show negative results for SIMO in the lactulose and glucose breath tests, with hydrogen and methane levels remaining below diagnostic thresholds. Figure (C,D) illustrate positive cases of Small Intestinal Bacterial Overgrowth on lactulose and glucose breath test, respectively. Figure (E,F) show positive cases of Intestinal Methanogen Overgrowth, both in the lactulose and glucose breath test. Abbreviations: SIBO: Small Intestinal Bacterial Overgrowth; IMO: Intestinal Methanogen Overgrowth; H_2_: hydrogen; CH_4_: methane.

## Abbreviations

The following abbreviations are used in this manuscript:

BT	Breath test
SIMO	Small intestinal microbial overgrowth
SIBO	Small intestinal bacterial overgrowth
IMO	Intestinal methanogen overgrowth
LBT	Lactulose breath test
GBT	Glucose breath test
CH_4_	Methane
H_2_	Hydrogen
BMI	Body mass index
PPV	Positive predictive value
NPV	Negative predictive value
