# Supplementary material for: Diagnostic Evaluation of Small Intestinal Microbial Overgrowth: A Cross-Sectional Comparison of Glucose and Lactulose Breath Tests
Source: J Clin Med. 2025 Dec 17;14(24):8920. doi: 10.3390/jcm14248920 (PMC12734151; doi:10.3390/jcm14248920)
Supplement: Supplementary file 1 [file jcm-14-08920-s001.zip › jcm-4007551-supplementary.pdf]

Table S1. Demographic and anthropometric characteristics of the study population.

	Total	Lactulose breath test	Glucose breath test	Combined breath tests
**Number of patients**	564	275	289	47
Women	426 (75.5%)	215 (78.2%)	211 (73.0%)	38 (80.9%)
Men	138 (24.5%)	60 (21.8%)	78 (27.0%)	9 (19.1%)
**Median age (years)**	52 (IQR: 38-65)	51 (IQR: 37-64)	52.50 (IQR: 39-65)	56 (IQR: 41-67.50)
**Median BMI (kg/m²)**	23.15 (IQR: 20.70-26.74)	23.10 (IQR: 20.82-26.23)	23.18 (IQR: 20.47-26.81)	24.30 (IQR: 21.77-27.73)

Abbreviations: BMI: body mass index; IQR: interquartile range.

Table S2. Prevalence of Small Intestinal Bacterial Overgrowth, Intestinal Methanogen Overgrowth and mixed type Overgrowth in patients who underwent both lactulose and glucose breath tests.

		Glucose breath test			
		**Negative**	**SIBO (peak in H_2)**	**IMO (peak in CH_4)**	**Mixed overgrowth (dual peak in H_2 and CH_4)**
**Lactulose breath test**	**Negative**	24	2	1	0
	**SIBO (peak in H_2)**	10	0	1	0
	**IMO (peak in CH_4)**	4	0	2	0
	**Mixed overgrowth (dual peak in H_2 and CH_4)**	3	0	0	0

Abbreviations: SIBO: Small Intestinal Bacterial Overgrowth; IMO: Intestinal Methanogen Overgrowth; SIMO: Small Intestinal Microbial Overgrowth; H_2: hydrogen; CH_4: methane.

Table S3. Differences in the prevalence of symptoms between negative and positive patients in the lactulose and glucose breath test subgroups.

	**Lactulose breath test (n = 142)**			**Glucose breath test (n =145)**			**Total (n = 287)**		
	**Pos. (n = 51)**	**Neg. (n = 91)**	**p-value†**	**Pos. (n = 27)**	**Neg. (n = 118)**	**p-value†**	**Pos. (n = 78)**	**Neg. (n = 209)**	**p-value†**
**Dyspepsia**	32 (62.7%)	60 (65.9%)	0.84	16 (59.3%)	75 (63.6%)	0.84	48 (61.5%)	135 (64.6%)	0.73
**Abdominal pain**	38 (74.5%)	62 (68.1%)	0.54	15 (55.6%)	76 (64.4%)	0.52	53 (67.9%)	138 (66.0%)	0.87
**Constipation**	14 (27.5%)	24 (26.4%)	1.00	7 (25.9%)	19 (16.1%)	0.36	21 (26.9%)	43 (20.6%)	0.32
**Diarrhea**	5 (9.8%)	23 (25.3%)	**0.045**	7 (25.9%)	26 (22.0%)	0.86	12 (15.4%)	49 (23.4%)	0.19
**Alternating bowel habits**	15 (29.4%)	31 (34.1%)	0.57	10 (37.0%)	39 (33.1%)	0.86	25 (32.1%)	70 (33.5%)	0.93
**Flatulence**	43 (84.3%)	80 (87.9%)	0.78	23 (85.2%)	98 (83.1%)	1.00	66 (84.6%)	178 (85.2%)	1.00

†: calculated using chi-square test.

Abbreviations: Pos.: positive patients; Neg.: negative patients.

Table S4. Differences in the prevalence of symptoms between patients with SIBO and those with IMO in the lactulose and glucose breath test subgroups.

	**Lactulose breath test (n = 47)**			**Glucose breath test (n = 25)**			**Total (n = 72)**		
	**SIBO (n = 32)**	**IMO (n = 15)**	**p-value†**	**SIBO (n = 9)**	**IMO (n = 16)**	**p-value†**	**SIBO (n = 41)**	**IMO (n = 31)**	**p-value†**
**Dyspepsia**	20 (62.5%)	11 (73.3%)	0.53	5 (55.6%)	11 (68.8%)	0.67	25 (61.0%)	22 (71.0%)	0.46
**Heartburn**	7 (21.9%)	3 (20.0%)	1.00	4 (44.4%)	6 (37.5%)	1.00	11 (26.8%)	9 (29.0%)	1.00
**Abdominal pain**	25 (78.1%)	11 (73.3%)	0.73	5 (55.6%)	8 (50.0%)	1.00	30 (73.2%)	19 (61.3%)	0.32
**Constipation**	7 (21.9%)	6 (40.0%)	0.30	0 (0.0%)	6 (37.5%)	0.06	7 (17.1%)	12 (38.7%)	0.06
**Diarrhea**	3 (9.4%)	2 (13.3%)	0.65	4 (44.4%)	3 (18.8%)	0.21	7 (17.1%)	5 (16.1%)	1.00
**Alternating bowel habits**	13 (40.6%)	2 (13.3%)	0.09	4 (44.4%)	5 (31.3%)	0.67	17 (41.5%)	7 (22.6%)	0.13
**Flatulence**	27 (84.4%)	13 (86.7%)	1.00	7 (77.8%)	14 (87.5%)	0.60	34 (82.9%)	27 (87.1%)	0.75

†: calculated using Fisher's exact test.

Abbreviations: SIBO: Small Intestinal Bacterial Overgrowth; IMO: Intestinal Methanogen Overgrowth.

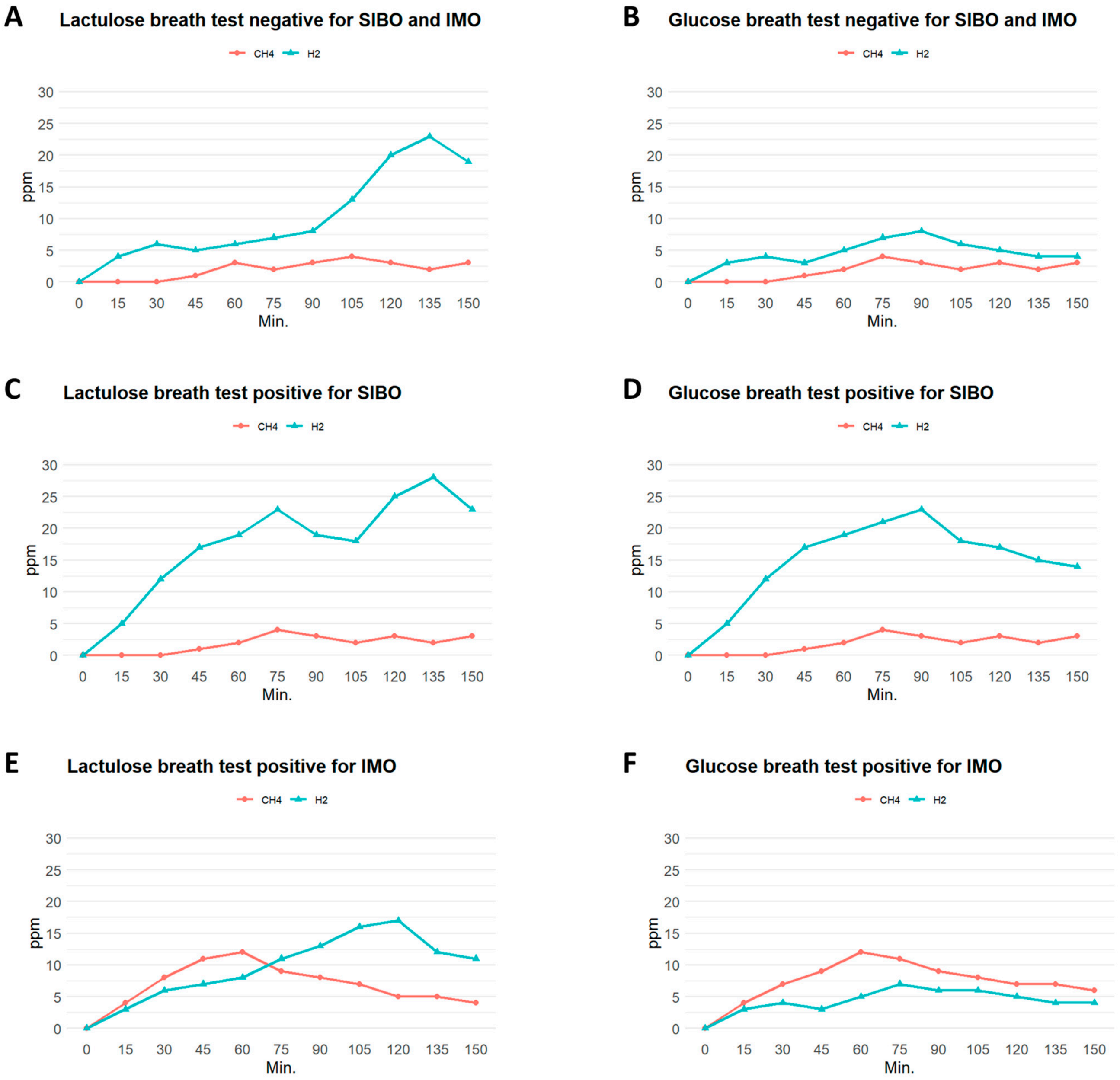

Figure S1. Graphic representation of the interpretation of the gas curves in lactulose breath test (LBT) and in glucose breath test (GBT). In LBT, the cut-off was defined as an increase of at least (≥) 20 parts per million (ppm) in H_2 (SIBO) or ≥10 ppm in CH_4 (IMO) above baseline by 90 minutes, while GBT was considered positive for SIMO with an increase of ≥10 ppm in H_2 (SIBO) or CH_4 (IMO) above baseline. **Figures A** and **B** show negative results for SIMO in the lactulose and glucose breath tests, with hydrogen and methane levels remaining below diagnostic thresholds. **Figures C** and **D** illustrate positive cases of Small Intestinal Bacterial Overgrowth on lactulose and glucose breath test, respectively. **Figures E** and **F** show positive cases of Intestinal Methanogen Overgrowth, both in the lactulose and glucose breath test. Abbreviations: SIBO: Small Intestinal Bacterial Overgrowth; IMO: Intestinal Methanogen Overgrowth; H_2: hydrogen; CH_4: methane.
